# Cold versus thermal neutron source: assessment of performance of the KWS-2 SANS diffractometer of the Jülich Centre for Neutron Science at the FRM II reactor

**DOI:** 10.1107/S1600576725006491

**Published:** 2025-08-13

**Authors:** Aurel Radulescu, Ralf Biehl, Aristeidis Papagiannopoulos

**Affiliations:** a Jülich Centre for Neutron Science at Heinz Maier-Leibnitz Zentrum Forschungszentrum Jülich GmbH Garching 85747 Germany; b Julich Centre for Neutron Science Forschungszentrum Jülich Jülich 52425 Germany; c Theoretical and Physical Chemistry Institute National Hellenic Research Foundation Athens 11635 Greece; Argonne National Laboratory, USA

**Keywords:** SANS, small-angle neutron scattering, neutron sources, protein structure, *McStas* simulation, neutron focusing lenses

## Abstract

The small-angle neutron scattering (SANS) diffractometer KWS-2 of the Jülich Centre for Neutron Science at the Heinz Maier-Leibnitz Zentrum, Garching, Germany, is a high-flux instrument based on the combination of a specially developed neutron guide system, which allows delivery of a high neutron intensity for a wide wavelength range λ between 2.8 and 20 Å to the instrument, and a versatile velocity selector, which allows an easy choice of wavelength spread Δλ/λ depending on whether the specific scientific goal is either improved resolution, *i.e.* Δλ/λ = 10%, or high intensity, *i.e.* Δλ/λ = 20%. Here we report an evaluation of KWS-2 performance by measurements and *McStas* simulations under the condition that only the thermal neutron source (TNS) is available at the FRM II reactor. According to the simulations, a flux decrease by a factor of 2.5 for λ = 2.8–3 Å and by a factor of 10 for λ ≥ 4.5 Å is expected if the reactor is operated only with the TNS compared with the established performance with cold neutrons provided by the cold neutron source. The flux decrease in TNS operation can be mitigated for λ ≥ 4.5 Å by using a low-resolution velocity selector which provides Δλ/λ = 20% for standard positioning and Δλ/λ = 35% when tilted at an angle ξ_i_ = −10° to the beam axis. According to measurements, this degradation of resolution for increasing intensity does not seem to be critical for the structural characterization of small biological morphologies, which would be one of the most investigated topics if only thermal neutrons were available at the FRM II. However, ordering effects in the scattering characteristics of lamellar soft or biophysical systems are barely observable when the resolution is relaxed to Δλ/λ ≥ 20%, which defines the performance limits of the experimental approach. Furthermore, by using MgF_2_ focusing lenses when working with large samples, an intensity gain on the sample of up to 12-fold is achieved while keeping the same *Q*_min_ resolution as with the standard pinhole mode, restoring the flux loss expected with thermal neutrons for λ ≥ 7 Å.

## Abstract 2

The performance of the small-angle neutron scattering (SANS) diffractometer KWS-2 is evaluated by measurements and *McStas* simulations under the condition that only the thermal neutron source is available at the FRM II reactor. This is compared with the established performance with cold neutrons provided by the cold neutron source.

## 1. Introduction

The small-angle neutron scattering (SANS) diffractometer KWS-2 (Radulescu *et al.*, 2012*a* ▸) operated by the Jülich Centre for Neutron Science (JCNS) at Heinz Maier-Leibnitz Zentrum (MLZ), Garching, Germany, is dedicated to the investigation of mesoscopic multi-scale structures and structural changes in soft condensed matter and biophysical systems. Following demands from the user community, it was repeatedly upgraded to enable the exploration of a broad *Q* range, between 2.0 × 10^−4^ and 1.0 Å^−1^, providing high neutron intensities and a tunable experimental resolution (Radulescu *et al.*, 2015 ▸; Houston *et al.*, 2018 ▸). Extension of the *Q* range up to *Q*_max_ = 2.0 Å^−1^ will soon be offered with 2D wide-angle detectors that will record neutrons scattered over a broad angular range up to θ_S_ = 50° (Radulescu *et al.*, 2023 ▸; Radulescu, 2024 ▸; Kang *et al.*, 2025 ▸). KWS-2 provides a high neutron flux on the sample as a result of the combination of a neutron guide system which was specially designed to transport high intensity to the instrument (Radulescu *et al.*, 2012*a* ▸) and a versatile velocity selector (Airbus, Germany) which enables an easy selection of the wavelength λ and wavelength spread Δλ/λ, depending on whether the specific scientific experiment demands either an improved resolution, thus Δλ/λ = 10%, or a high intensity, hence Δλ/λ = 20% (Houston *et al.*, 2018 ▸). The resolution can be further improved down to Δλ/λ = 2%, in a range where no velocity selector can compete (Radulescu *et al.*, 2015 ▸; Radulescu, 2024 ▸), by using the time-of-flight (TOF) mode with a versatile chopper providing a variable slit opening and a variable frequency, to match the optimal TOF conditions depending on the wavelength λ and the sample-to-detector distance *L*_D_ used. The high-flux option enables scientific opportunities in the field of structural investigation of small soft-matter and biological systems, aiming at time-resolved SANS investigations of fast kinetical processes with a time resolution of a few tens of ms (Radulescu *et al.*, 2012*b* ▸; Zinn *et al.*, 2012 ▸). It also provides the conditions for optimal utilization of beam time by acquiring data in a shorter time than is possible on many other SANS diffractometers. The installation of robotic elements and an automated sample changer at the sample position (Radulescu *et al.*, 2023 ▸) allows a continuous supply of samples to the instrument and the merging of experiments from different user teams when similar experimental conditions are required, contributing even more to the efficient use of beam time.

Due to current technical problems (Pichlmaier *et al.*, 2022 ▸), the FRM II reactor will be operated temporarily without a cold neutron source (CNS). Instruments at the MLZ that are designed to operate with thermal or hot neutrons remain unaffected by this change. However, instruments designed for cold neutrons will experience a drastic change in flux and operating conditions, and may not operate at all, depending on the instrument profile and the neutron guide and monochromator features. Therefore, the evaluation of KWS-2 performance under the new conditions where only the thermal neutron source (TNS) is available at the FRM II reactor compared with the established cold neutron performance (Radulescu *et al.*, 2012*a* ▸; Radulescu *et al.*, 2012*b* ▸; Houston *et al.*, 2018 ▸; Balacescu *et al.*, 2021 ▸; Radulescu *et al.*, 2023 ▸) is of great importance for the user community. This will allow the optimization and adjustment of the provisional scientific goals at the instrument until the normal operating conditions of the FRM II reactor are restored.

This paper reports the optimal experimental settings, test measurements and expected performance in terms of neutron intensity on the sample and scattering data quality when using λ = 2.8–7.0 Å neutrons at the SANS diffractometer KWS-2 in combination with *McStas* simulations of the flux distributions at different points along the neutron guide system and the instrument. The short neutron wavelengths λ < 4.5 Å are available at KWS-2 through the Airbus velocity selectors when operated in a tilted position with an angle of ξ_i_ = −10° with respect to the beam axis, as well as through the neutron guide characteristics. All these elements were taken into account in the *McStas* simulation of the KWS-2 instrument with a cold source and a thermal source of the FRM II reactor. The quality of the SANS data in the medium- and high-*Q* range for experiments with λ = 3 Å was assessed by measuring the proteins bovine serum albumin (BSA) and alcohol de­hydrogenase (ADH) at different volume fractions in buffer solution. Additionally, a system consisting of mixed liposomes of dipalmitoylphosphatidylcholine (DPPC) and gradient(pseudo-diblock) poly(2-methyl-2-oxazoline)-*grad*-poly(2-phen­yl-2-oxazoline) (MPOx2) copolymers in D_2_O, characterized by vesicular populations of different lamellarity, measured at KWS-2 with λ = 4.5 Å (Papagiannopoulos *et al.*, 2021 ▸), was analyzed for different instrument resolutions to check the observability limit of sharp scattering features, while the resolution was relaxed to enhance the intensity in the case of using only thermal neutrons at KWS-2.

Further optimizations aimed at providing a high intensity on the sample for λ ≥ 7 Å while keeping the resolution as for the standard mode (*Q*_min_ = 1 × 10^−3^ Å^−1^ and Δλ/λ = 10%) by using focusing lenses and large sample size (up to 5 cm in diameter) or relaxing the resolution (Δλ/λ > 20%) for λ ≥ 4.5 Å are also discussed, on the basis of experiment and simulation results. The working conditions in the thermal neutron regime of the FRM II reactor are discussed in detail in relation to the normal operating conditions with cold neutrons.

## 2. Experimental

### 2.1. Proteins and polystyrene particles in solution

BSA and ADH proteins were purchased from Sigma–Aldrich. The protein powders were dissolved in a buffer solution composed of 10 m*M* NaH_2_PO_4_/Na_2_HPO_4_ and 100 m*M* NaCl in D_2_O (99.9 at.% D) at pH 7.5. The freshly prepared protein solutions were dialyzed against D_2_O buffer with an excess volume of 100 times in order to virtually remove any exchangeable protons from the protein. In order to remove aggregates, the solutions were filtered through a 0.2 µm membrane filter and centrifuged for several hours. The protein concentration was adjusted in different samples to 5 and 20 mg ml^−1^ by buffer dilution. Details of sample preparation can be found in the work of Ameseder *et al.* (2018 ▸) and Monkenbusch *et al.* (2015 ▸). The samples of the protein solutions as well as of the dialyzate buffer solutions were transferred in banjo-type quartz Hellma cuvettes with 1 mm sample path and sealed with paraffin wax at the top.

Phospho­lipid films were formulated by mixing in a 9:1 *v*/*v*% ratio in chloro­form DPPC (Avanti Polar Lipids Inc.) and gradient pseudo-diblock MPOx2, which was synthesized via cationic polymerization as described extensively elsewhere (Papagiannopoulos *et al.*, 2021 ▸). Following slow chloro­form removal, desiccated films were obtained, which were subsequently hydrated in D_2_O yielding DPPC–MPOx2 vesicles with different lamellarity, as described by Papagiannopoulos *et al.* (2021 ▸).

Polystyrene size standard spherical particles (PS) with a radius of *R* = 150 Å and a size polydispersity (standard deviation divided by the mean) of σ_*R*_ ≅ 8% were purchased from ThermoFisher Scientific in aqueous solution.

### 2.2. SANS experiments

SANS measurements of the BSA and ADH protein solutions were carried out at the KWS-2 SANS diffractometer (Radulescu *et al.*, 2012*a* ▸; Radulescu *et al.*, 2012*b* ▸; Radulescu *et al.*, 2015 ▸; Houston *et al.*, 2018 ▸; Balacescu *et al.*, 2021 ▸; Radulescu *et al.*, 2023 ▸) in the high-*Q* regime using the main detector positioned at two detection distances after the sample, namely *L*_D_ = 1.25 and 4 m, and a wavelength λ = 3 Å with Δλ/λ = 14%, as delivered by the Airbus velocity selector used in tilted configuration with an angle ξ_i_ = −10° with respect to the beam axis (Houston *et al.*, 2018 ▸; Radulescu, 2024 ▸). The data were normalized to the upstream monitor, corrected on a pixel-to-pixel basis for the empty cuvette contribution, detector sensitivity and instrument background following the typical SANS reduction procedure (Radulescu *et al.*, 2016 ▸), calibrated in absolute units (cm^−1^) using the Plexiglas secondary standard (Houston *et al.*, 2018 ▸), and radially averaged to deliver 1D data. The correction for the buffer scattering contribution was subsequently applied on 1D corrected and calibrated protein data. Measurements on lamellar vesicles are described by Papagiannopoulos* et al.* (2021 ▸).

Measurements with lenses in the enhanced intensity mode were carried out on PS particles (*R* = 150 Å) in a 0.1 *v*/*v*% solution in D_2_O by using an entrance aperture of 50 × 50 mm, as for the pinhole mode, at *L*_C_ = 20 m, with all 26 MgF_2_ parabolic lenses in beam (Radulescu *et al.*, 2012*a* ▸). The square sample aperture was gradually increased from 10 × 10 mm, as for the pinhole mode, to 30 × 30 mm and finally to a round shape with diameter ϕ = 50 mm. Lenses in the evacuated lens-chamber were used in the cold state at a temperature of *T* = 70 K, achieved by using a cold head (VeriCold Technologies GmbH, Germany) driven by a Coolpak compressor (Leybold GmbH, Germany).

## 3. Simulations

The simulation of the KWS-2 instrument and its performance in terms of intensity transported from the neutron source downstream to the sample position was performed with the *McStas* program package (Willendrup *et al.*, 2004 ▸) in two situations: either with the CNS or with the TNS of the FRM II reactor (Carpenter & Loong, 2015 ▸). The KWS-2 neutron guide system and instrument, including the CNS, the guide characteristics (profile, geometrical and coating parameters *etc*.), the velocity selector and the collimation system (position and size of the apertures), were used as in previous simulations (Radulescu *et al.*, 2012*b* ▸; Radulescu & Ioffe, 2008 ▸), covering the main features of the beamline. The TNS used in the simulations considering the case of absence of the CNS was calculated and provided by Röhrmoser (2021 ▸).

## 4. Results and discussion

### 4.1. Simulations of the CNS and TNS cases

**Figure 1:**
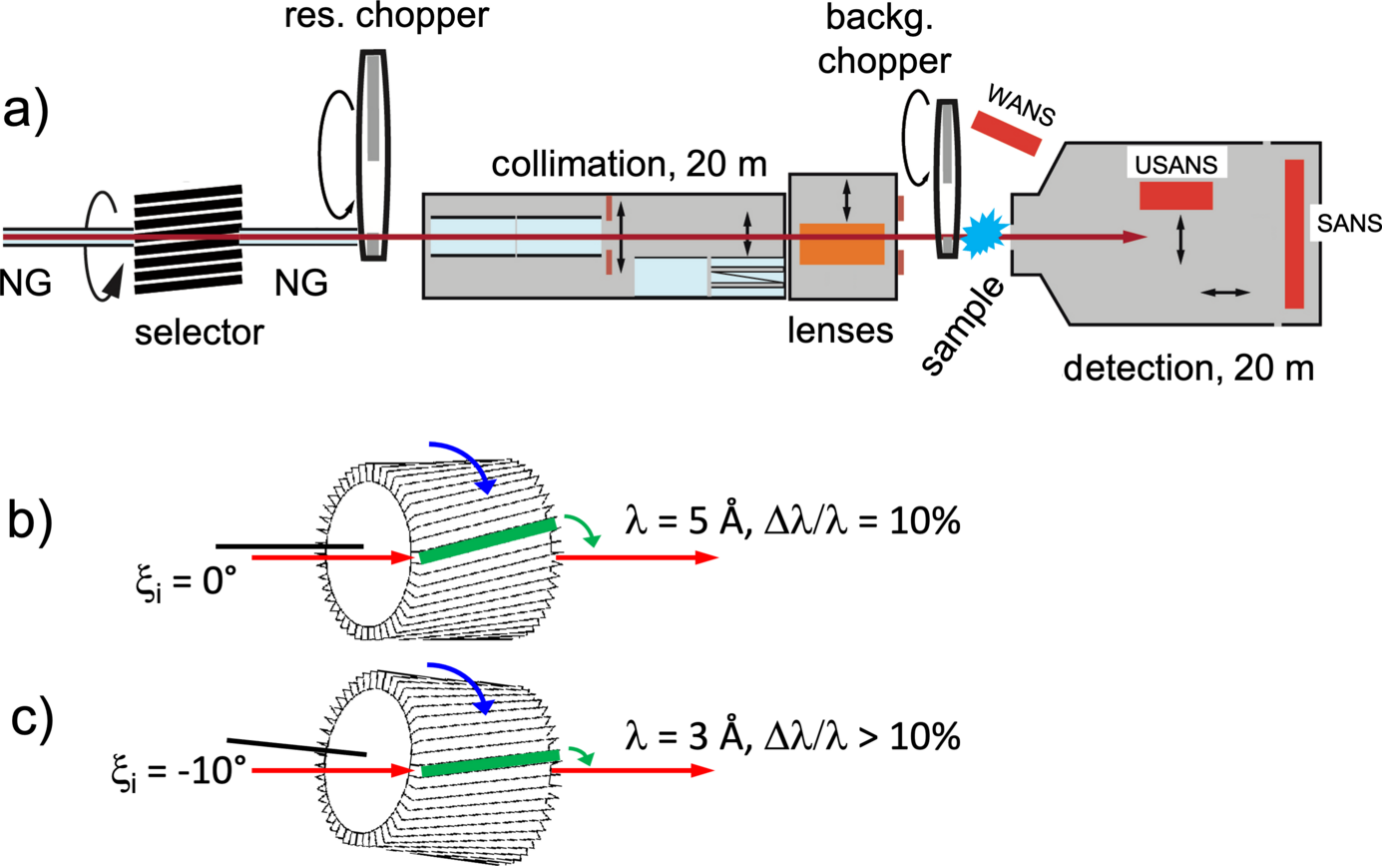
Layout of the KWS-2 instrument (*a*) (credit MLZ) with detailed presentation of the velocity selector used in a parallel positioning to the beam axis (*b*) or tilted with an angle ξ_i_ = −10° with respect to the beam axis (*c*).

KWS-2 is equipped with a velocity selector (Airbus, Germany) that plays the role of monochromator and provides the instrument with neutrons of the needed wavelength λ and wavelength resolution Δλ/λ for fulfilling the experimental goals (Fig. 1 ▸). Since the velocity selector can be operated at different velocities and inclination angles to the beam direction, neutrons with λ = 2.8 and 10.0 Å can be used complementarily to the typically used wavelengths λ = 4.5 or 7.0 Å, to cover an extended *Q* range in pinhole mode from 0.001 to 1.0 Å^−1^. Tilting of the selector with respect to the beam axis enables the shift of λ_min_ from 4.5 Å in the standard configuration to 2.8 Å, at the highest rotation speed of 470 rps (revolutions per second).

However, tilting of the velocity selector is a two-way approach: the provision of shorter wavelengths compared with the standard configuration is accompanied by the worsening of the Δλ/λ wavelength spread and the limitation of the largest wavelength available. Thus, the velocity selector in operation at KWS-2 delivers a monochromatic λ in the range 4.5–20 Å with Δλ/λ = 10% when it is operated in the standard configuration, with its axis parallel to the beam axis and with rotation speeds from 130 to 470 rps. When used in a tilted configuration with the tilting angle ξ_i_ = −10° with respect to the beam axis, the λ range is shifted to 2.8–7 Å with Δλ/λ between 14% and 19.5%, for the same range of rotation speed. Larger values than λ = 7 Å in the tilted configuration are prohibited due to vibrations from resonance frequencies of the selector. On demand, to increase the intensity on the sample for a relaxed resolution, a Δλ/λ = 20% velocity selector in standard configuration may be installed and used in either a tilted or a parallel positioning with respect to the beam axis. This selector was part of the standard configuration of the instrument until the middle of 2017 and provided a wavelength resolution of Δλ/λ = 22.5% for λ = 3 Å and Δλ/λ ≅ 35% for λ ≥ 4 Å when it was used in a tilted configuration with an angle ξ_i_ = −10° with respect to the beam axis (Houston *et al.*, 2018 ▸). Due to the safety elements in connection with the tilting procedure of the velocity selector in operation, there is no elevator installed at the position of the velocity selector of the KWS-2 that would allow a quick change between a high-resolution selector (10%) and a high-intensity selector (20%) if required. However, in our experience, this operation can be performed relatively quickly, within a couple of hours, if a spontaneous decision is made in this regard, due to the presence of fixation and alignment elements on the selector platform. In addition, careful planning of experiments allows the use of a dedicated selector over a longer period of time, with replacement with a more suitable selector during reactor breaks or scheduled maintenance at the instrument.

The characteristics of the neutron guide system that supplies KWS-2 with neutrons have been discussed in detail in previous publications (Radulescu *et al.*, 2012*a* ▸; Radulescu *et al.*, 2012*b* ▸; Radulescu & Ioffe, 2008 ▸). Here we only briefly mention that, starting from the FRM II source, the NL3 guide (50 × 170 mm) is split into two sub-guides after about an 8 m straight section. One of these, NL3a (50 × 110 mm), extends horizontally curved with a radius *R* = 460 m over a length of 16.3 m and is double-channeled, so equipped with a central vertical splitting wall. It is then split again with the upper section (50 × 50 mm) continuing horizontally curved with the same curvature radius and a double channel for about 2.3 m up to the KWS-2 instrument shutter, in the casemate of the neutron guide hall. The other parts of the NL3 and NL3a guides feed the KWS-1 and the KWS-3 instruments. From the KWS-2 instrument shutter, the neutron guide continues further, horizontally straight but vertically S-shaped (11 m + 11 m) with a curvature of the S-halves of *R* = 800 m, and reaches the entrance of the collimation system of KWS-2. The spatial intensity distribution of neutrons emerging from a curved guide is asymmetric (Radulescu *et al.*, 2012*b* ▸). As explained by Radulescu & Ioffe (2008 ▸), while working with a shorter collimation length *L*_C_, further neutron guides are thus placed in beam in the collimation system up to the entrance (collimation) aperture, which is placed closer to the sample; those guides play a role in homogenizing the spatial intensity distribution along the collimation system of KWS-2.

However, the neutrons entering the instrument at a very high angle cannot be transported up to the sample position regardless of whether a pair of apertures for the pinhole geometry or further neutron guide segments are active in the collimation system, as shown by Radulescu & Ioffe (2008 ▸). As reported by Radulescu *et al.* (2012*b* ▸), the flux at the sample position shows an 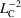 behavior that represents the ideal dependence law, demonstrating that all neutrons fulfilling the optical conditions are transported to the sample position in all *L*_C_ conditions.

The horizontally curved guide in the tunnel ensures that there is no line of sight through the guide, which suppresses γ radiation and fast neutron contamination of the beam. The use of the S-shaped guide in the casemate and neutron guide hall, up to the entrance of the instrument, is dictated by the predefined dimensions, especially the height, of KWS-2, which was built at the FRJ-2 reactor in Jülich at the end of the 1980s for a higher beam position than that at the FRM II reactor in Garching. A readjustment of the instrument geometry at the time it was moved from the FRJ-2 reactor to FRM II was excluded due to costs and time delay considerations. The velocity selector is placed in the center of the S-guide, with its axis following the inclination of the neutron beam axis at this point, in a gap of about *L*_sel_ = 0.35 m made in the neutron guide for this purpose (Radulescu & Ioffe, 2008 ▸). The selector is positioned so that the neutron window is at ‘3 o’clock’ in the direction of the neutrons flying towards the instrument (Radulescu *et al.*, 2012*b* ▸). In addition, the double-disc resolution chopper of the instrument is placed towards the end of the S-guide, just in front of the instrument, where two narrow gaps of *L*_chopp_ = 0.02 m are made in the guide for the two chopper discs (Radulescu *et al.*, 2015 ▸). The coating of the different sides of the neutron guide sections was optimized using *McStas* and *VITESS* (Zendler *et al.*, 2014 ▸) simulations, depending on the geometry and shape of these sections. The main goal of the optimization procedure was the transportation of high intensities of cold neutron spectrum up to the sample position and achievement of a cut-off of the S-guide at low λ values in order to enable a good intensity on the sample when using the tilted velocity selector; simulated and measured fluxes for different configurations have already confirmed that this goal has been met (Radulescu & Ioffe, 2008 ▸; Radulescu *et al.*, 2012*b* ▸; Houston *et al.*, 2018 ▸).

**Figure 2:**
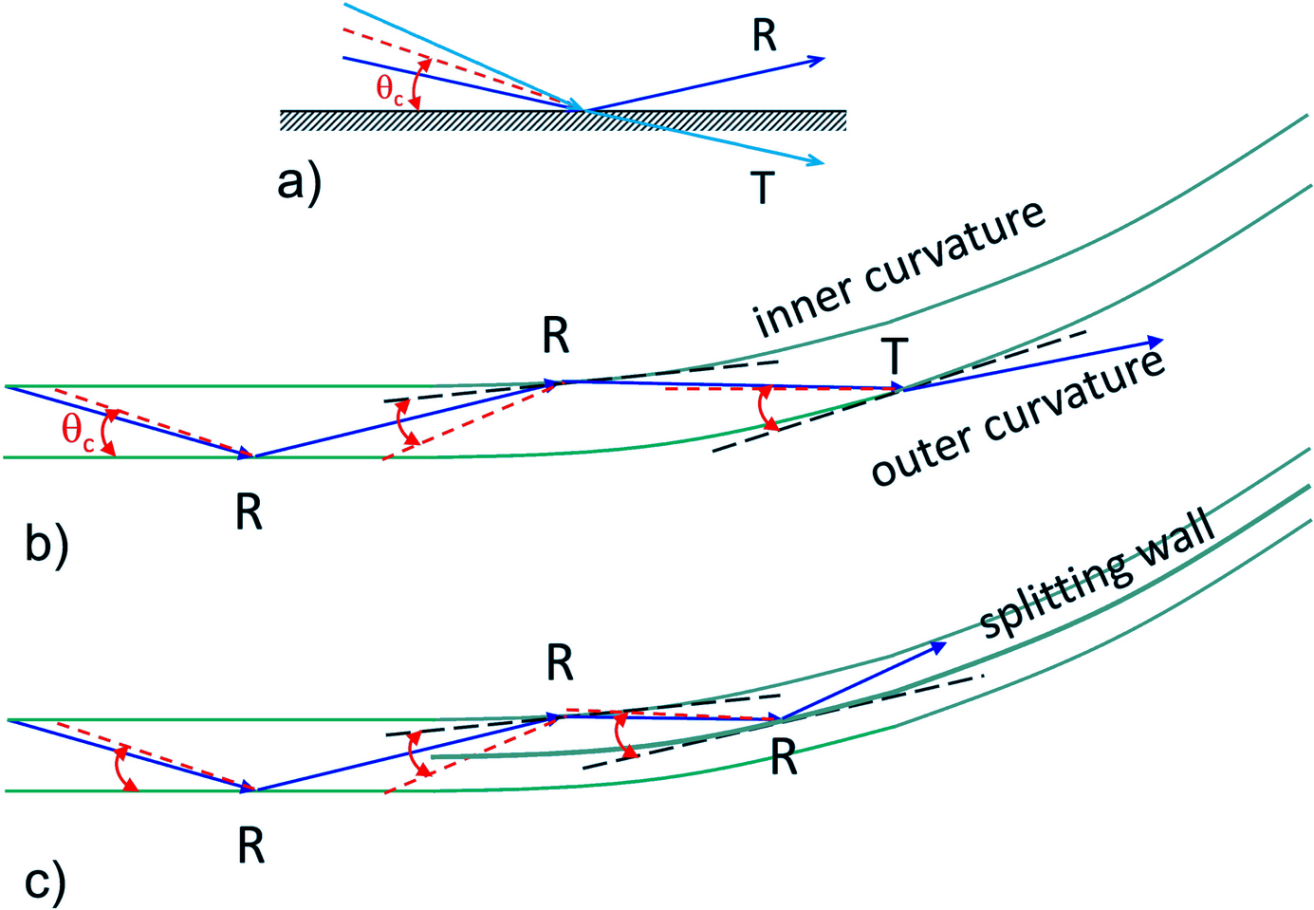
Schematic representation of the processes taking place during transportation of cold neutrons through a neutron guide: (*a*) reflection (R) and transmission (T) of neutrons when hitting the neutron guide wall depending on the incoming angle versus critical angle of the guide coating θ_c_; (*b*) processes along a curved guide and (*c*) processes along a curved guide with inner splitting wall. The black dashed line represents the tangent plane to the guide curvature at the impact point, while the red dashed line indicates the trajectory corresponding to the critical angle at the impact point, which is depicted by the red double arrows.

The cut-off of a neutron guide refers to the rapid drop in brightness at lower wavelengths caused by the upstream guide’s curvature combined with the wavelength dependence of the critical angle θ_c_ of reflection from the coating of the guide. The critical angle θ_c_, which is the maximum angle of the total reflection [Fig. 2 ▸(*a*)], characterizes the efficiency of a neutron guide in the transport of cold neutrons over long distances. All neutrons falling onto the neutron guide plane under this angle are reflected. Otherwise, they are transmitted through the coating and usually absorbed in the boron-rich guide wall, and hence lost from the transported beam along the guide. This angle is a characteristic of the reflecting property of the coating materials of the inner faces of a neutron guide and is given by
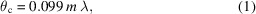
with θ_c_ given in degrees and λ in Å (Böni *et al.*, 2010 ▸). For ^nat^Ni, typically used for coating the straight neutron guides, the parameter *m* is equal to 1. To reflect neutrons at higher angles one could use ^58^Ni coating, for which *m* = 1.18, or use supermirrors, which today can be produced up to *m* = 7 (Schanzer *et al.*, 2016 ▸). High-*m* coatings are used in the case of curved neutron guides when the angle of incoming neutrons onto the inner face of the outer curvature side of the guide (*i.e.* the concave face) is higher than the θ_c_ due to the guide curvature [Fig. 2 ▸(*b*)]. Because θ_c_ varies with λ, short-wavelength neutrons are more affected by the guide curvature than long-wavelength neutrons; therefore, the intensities of lower-λ neutrons decrease more than those of longer-λ neutrons when such guides are used. To alleviate this effect, the concave faces of the curved guides are typically coated with a material of higher *m* than the coatings of the other faces, to increase θ_c_ and compensate for the curvature effect on the angle of incoming neutrons onto the guide plane. Another way to help transport higher intensities of lower-λ neutrons through a curved guide is to channel the guide simultaneously with using a higher *m* for the concave guide face, in order to ensure a lower angle for the incoming neutrons onto the concave faces than θ_c_, as depicted in Fig. 2 ▸(*c*). All these guide characteristics were followed in the current simulations by examining the flux distributions at different points along the KWS-2 guide system.

**Figure 3:**
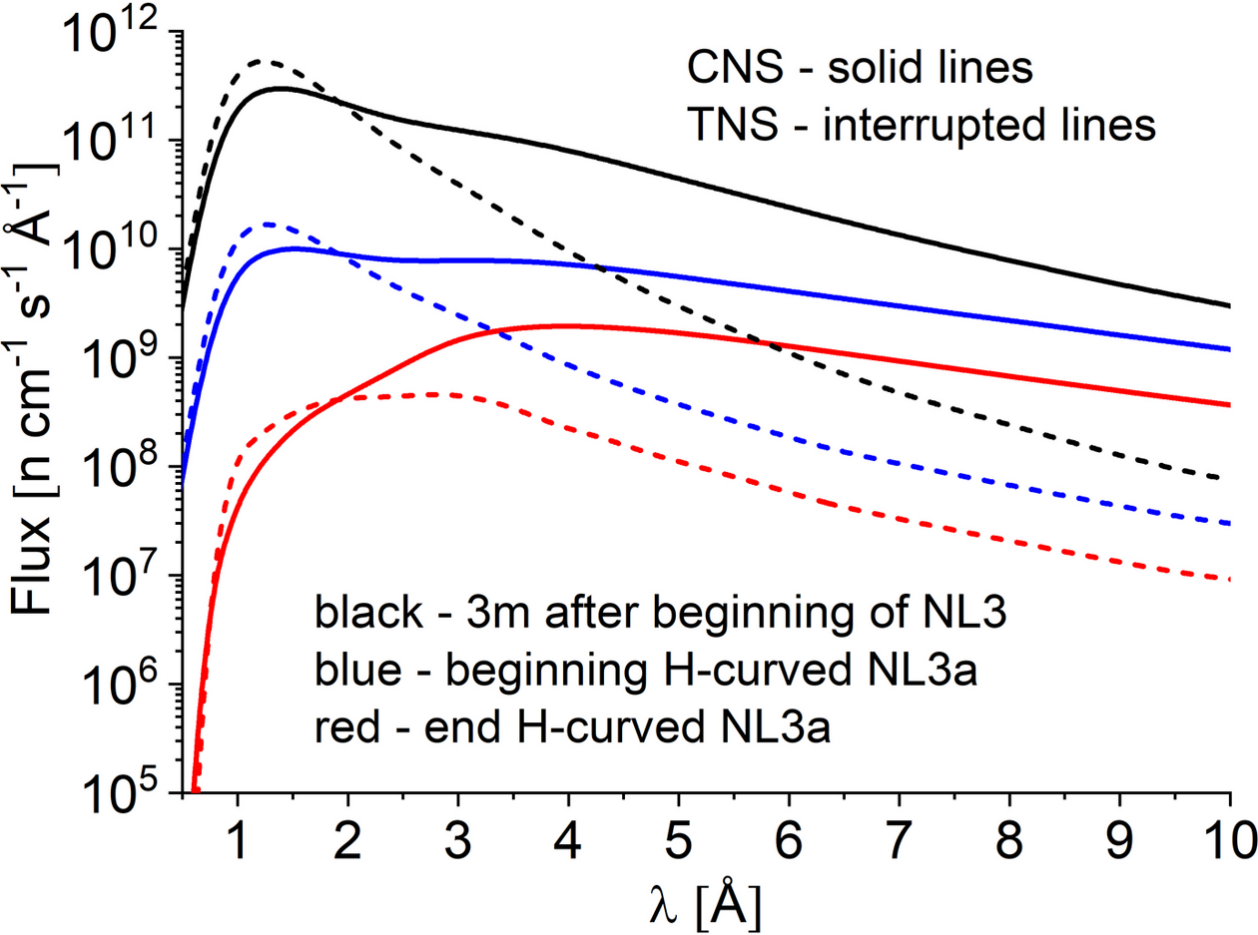
Simulated wavelength distribution of the neutron flux at different positions along the NL3 and NL3a guide system of KWS-2 up to the instrument shutter for the two reactor sources considered (see the text for detailed explanation).

Fig. 3 ▸ presents the flux distributions simulated by *McStas* at different positions along the NL3–NL3a guide system up to the instrument shutter, thus before the start of the vertical S-shaped guide of KWS-2, considering either the CNS (the continuous lines) or the TNS (the interrupted lines) of the FRM II reactor. The role of the CNS is obvious: much higher intensities of long-wavelength neutrons, λ > 2.2 Å, are delivered to the neutron guide system by the CNS compared with the case of the TNS, when very short wavelength neutrons are delivered with much higher intensities. The flux distributions simulated at 3 m from the entrance of NL3 resemble the trend and features reported for the neutron sources at FRM II (Carpenter & Loong, 2015 ▸) since at this position the effect of the neutron guide is minimal.

The features of the flux distributions are maintained with only the intensity decreasing as the beam advances along the straight guide (blue curves in Fig. 3 ▸). In contrast, the horizontally curved double-channeled guide causes a drastic decrease of intensity for low wavelengths λ < 3 Å, due to the effect discussed above (red curves in Fig. 3 ▸). To still transport as high an intensity as possible in the range λ = 2–3 Å, an *m* = 3 supermirror coating of the concave face of the guide was used, while the other faces were coated with *m* = 2 supermirrors.

**Figure 4:**
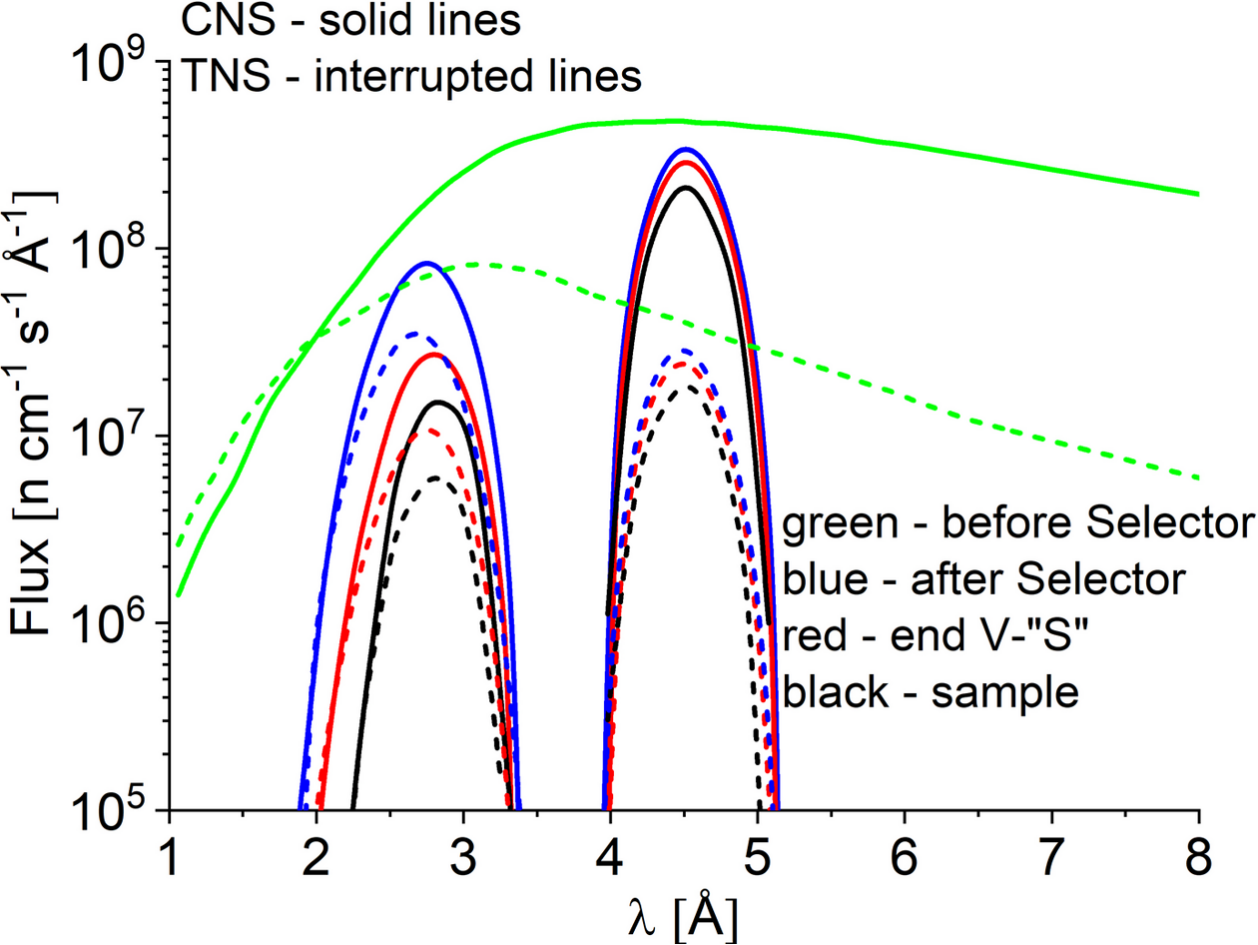
Simulated wavelength distribution of the neutron flux at different positions along the vertical NL3a–o ‘S’-guide and at the sample position of KWS-2 (see the text for detailed explanation), for the two reactor sources considered.

The flux distribution in the middle of the vertically S-shaped guide (Fig. 4 ▸), just before the velocity selector, shows a general decrease of intensities for all wavelengths, which is however more accentuated for shorter wavelengths, λ < 3 Å. The trend is similar in both the CNS and TNS cases. For the TNS case, the higher intensity in the range λ = 1–2 Å is further maintained, although this is not particularly important for the velocity selector fed SANS instrument. After the velocity selector the flux distributions are of course much narrower. They show in linear–linear presentation a triangle-like behavior, centered at a value corresponding to the monochromatic wavelength λ with the characteristic Δλ/λ as delivered by the velocity selector according to its setting (*i.e.* velocity, number of twisted blades, gap between the twisted blades, length, twisting angle, tilting angle *etc*.) (Radulescu *et al.*, 2012*b* ▸).

For a monochromatic beam with λ = 4.5 Å and Δλ/λ = 10% delivered by the velocity selector positioned parallel to the beam axis and running at the highest rotation speed, the intensity decreases only weakly further along the neutron guide and the instrument, as observed at the end of the S-guide and at the sample position (Fig. 4 ▸), without any change in the central wavelength of the Gaussian distribution. The same trend is shown for both the CNS and TNS cases, although with the TNS the intensity is much lower than with the CNS, as expected. The simulated flux at the sample position for a collimation length *L*_C_ = 2 m (maximum flux) when CNS was used is about 1 × 10^8^ n cm^−2^ s^−1^, while in the case of the TNS the simulated flux is only about 1 × 10^7^ n cm^−2^ s^−1^. For the monochromatic neutron beam delivered by the tilted velocity selector (ξ_i_ = −10° against the beam axis), again at the highest rotation speed (λ < 3 Å), the maximum of the beam distribution is shifted smoothly towards larger wavelengths in advancing from the velocity selector position to the sample position, where it reaches λ = 2.8 Å with Δλ/λ = 14%. The intensity decreases significantly when reaching the end of the S-guide and further at the sample position, unlike the case of the beam with λ = 4.5 Å. The maximum of the intensity distribution simulated in the case of using the TNS also migrates smoothly towards higher λ values in going from the velocity selector to the sample position, and the intensities are lower than those obtained for the CNS case, although the decrease is not as large as for λ = 4.5 Å. The simulated flux at the sample position, again for a collimation length *L*_C_ = 2 m (maximum flux), was about 3.5 × 10^6^ and 8.5 × 10^6^ n cm^−2^ s^−1^ for the case of the TNS and CNS, respectively. Thus, reduction factors of 10 and 2.5 are observed in the neutron flux at the sample position for λ = 4.5 and 2.8 Å, respectively, when the TNS is used instead of the CNS. This again underlines the role of a CNS at the reactor providing high flux for the cold neutron instruments such as the SANS diffractometers. On the other hand, the reduction factor in the case of neutrons with λ = 2.8 Å indicates that short-wavelength neutrons from the cross-over region between the CNS and TNS (Fig. 3 ▸) are less affected when no CNS is in operation at the reactor. The variation of the maximum of the distribution towards higher λ values for very short wavelength neutrons is an effect of the properties of the neutron guide: the gaps in the neutron guide system (selector, end of the guide/input of the instrument) and changes in shape, orientation and coating seem to be responsible for this effect observed for neutrons with λ = 2.8 Å, near the cut-off of the vertically curved guide of KWS-2, as described above. The neutrons with such a low wavelength are reflected at a lower θ_c_ than the neutrons with λ = 4.5 Å; therefore, they are more sensitive to changes in the geometry along the curved guide system and the collimation path of the instrument. This effect also explains the larger intensity decrease between the selector position and the sample position observed for λ = 2.8 Å compared with only a weak intensity decrease observed for λ = 4.5 Å for a given neutron source configuration. For λ = 4.5 Å, a high intensity is transported to the sample position despite all gaps and changes in the coating specifications of the guide, due to an optimized choice of coating, as aimed for during the design of the neutron guide system (Radulescu & Ioffe, 2008 ▸; Radulescu *et al.*, 2012*b* ▸).

### 4.2. Improvements in neutron flux at the sample position in the TNS regime

The CNS of the FRM II has been damaged, as was recently established (Pichlmaier *et al.*, 2022 ▸); it has already been removed and work is underway to produce a new CNS. To mitigate the serious disadvantages for the user community, it is planned to provide users with only thermal neutrons until the new CNS is installed and put into operation towards 2028, according to current estimations. The instruments for cold neutrons will be affected to varying degrees by this temporary special operating mode of the reactor, depending on the flux distribution with which they are fed. For KWS-2, the use of neutrons with λ ≥ 2.8 Å at a lower flux when only TNS is operated compared with the normal operation mode will still be possible to a large extent. Without the CNS at the FRM II reactor, the KWS-2 instrument will be at the same flux condition as before 2006, when the instrument operated at the former FRJ-2 reactor. These flux conditions are also fairly comparable to those met at SANS instruments operating at various neutron sources such as SINQ at the Paul Scherrer Institut, Switzerland (Kohlbrecher & Wagner, 2000 ▸), the Opal reactor at ANSTO, Australia (Wood *et al.*, 2018 ▸), the JRR-3 reactor of JAEA, Japan (Kumada *et al.*, 2023 ▸), HFR at ORNL, USA (Melnichenko, 2016 ▸), or NIST, USA (Barker *et al.*, 2022 ▸), as can be concluded by consulting the respective references. However, the research topics to be investigated at KWS-2 during the period without the CNS will have to be revised. Priority will be given to studies of small morphologies and shorter characteristic sizes, such as *e.g.* biological structures (proteins, membranes), small polymer micelles or micelle assemblies, semi-crystalline polymer films *etc*., which require shorter *L*_C_ and *L*_D_ in the instrument setup to cover these sizes and characteristic lengths in real space.

Gels, networks, large micelles and colloidal particles are characterized by large correlation lengths and characteristic sizes, and typically require *L*_D_ ≥ 8 m and λ ≥ 4.5 Å for a complete structural characterization. Studies of these systems will require an adjustment of the experimental setup to achieve an intensity on the sample that allows measurements under such conditions to be carried out over an acquisition time similar to that of the CNS regime. This can be achieved by using dedicated velocity selectors to relax the Δλ/λ wavelength resolution in exchange for flux gain. Another possibility is to use focusing lenses and a larger sample size. Focusing lenses placed in front of the sample aperture offer the possibility of maintaining the same *Q*_min_ resolution as in the pinhole mode, allowing an enhanced intensity by increasing the beam size on the sample and hence the sample size.

#### 4.2.1. Effect of a low-resolution velocity selector

**Figure 5:**
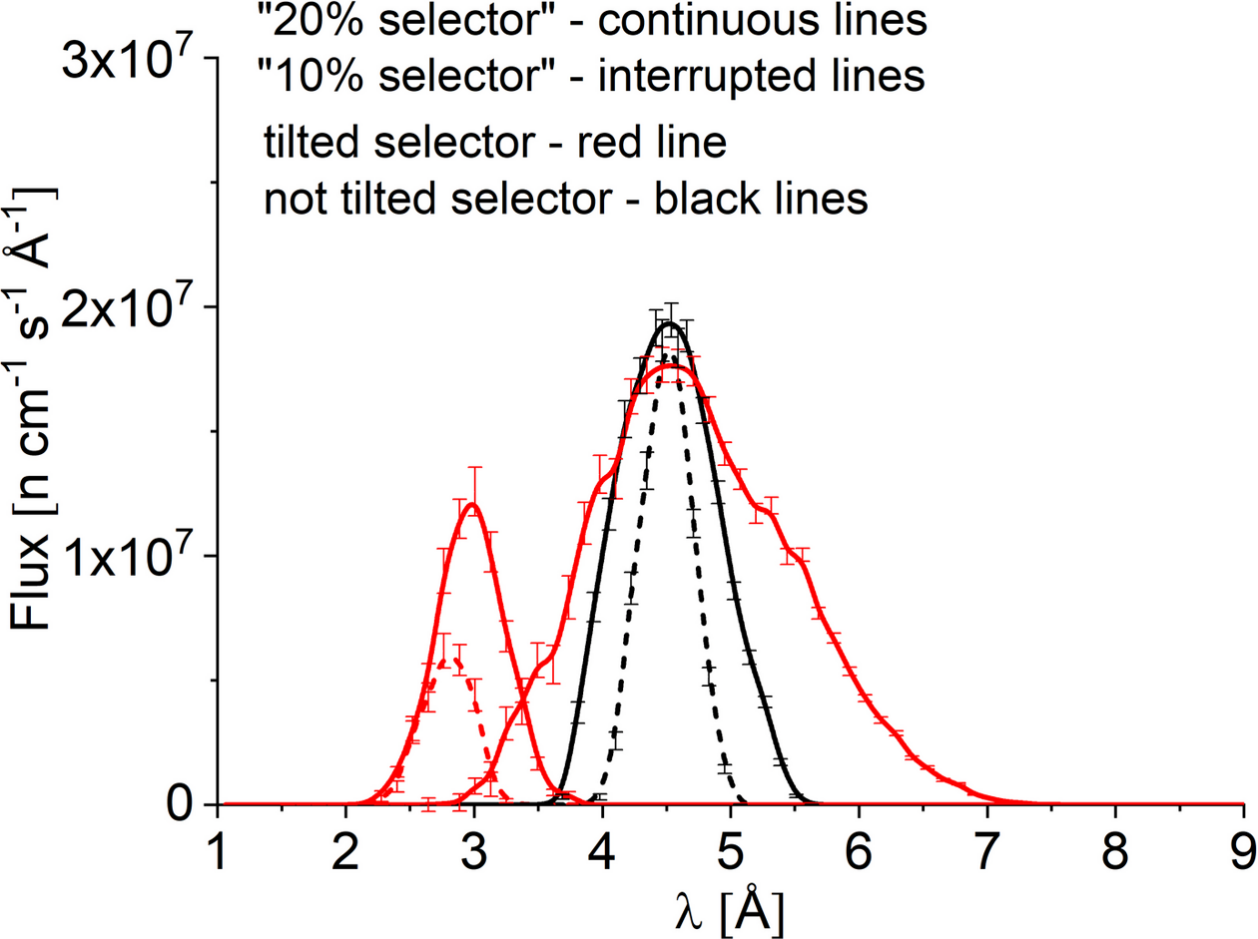
Simulated wavelength distribution of the neutron flux at the sample position of KWS-2 for the TNS and different velocity selectors with different inclinations against the beam direction considered (see text for details).

An increase in flux when only the TNS is operational at FRM II is considered by operating the instrument with a low-resolution velocity selector which provides in a standard configuration, while running parallel with the beam axis, a Δλ/λ = 20% wavelength distribution. *McStas* simulation results (Fig. 5 ▸) show that a significant increase in flux at the sample position can be obtained in this case compared with the conventionally used Δλ/λ = 10% selector. An increase by a factor of 2 can be obtained for neutrons with λ = 4.5 Å (selector not tilted) and by about 2.45 for neutrons with λ = 3 Å (tilted selector). Tilting a ‘20%’ selector used at lower speed (260 rps for the velocity selectors used at KWS-1 and KWS-2 SANS diffractometers of JCNS in Garching, Germany) will produce neutrons with λ = 4.5 Å characterized by Δλ/λ = 35%, which allows an increase by a factor of 3.5 in flux compared with the standard mode in the TNS regime (Fig. 5 ▸).

**Figure 6:**
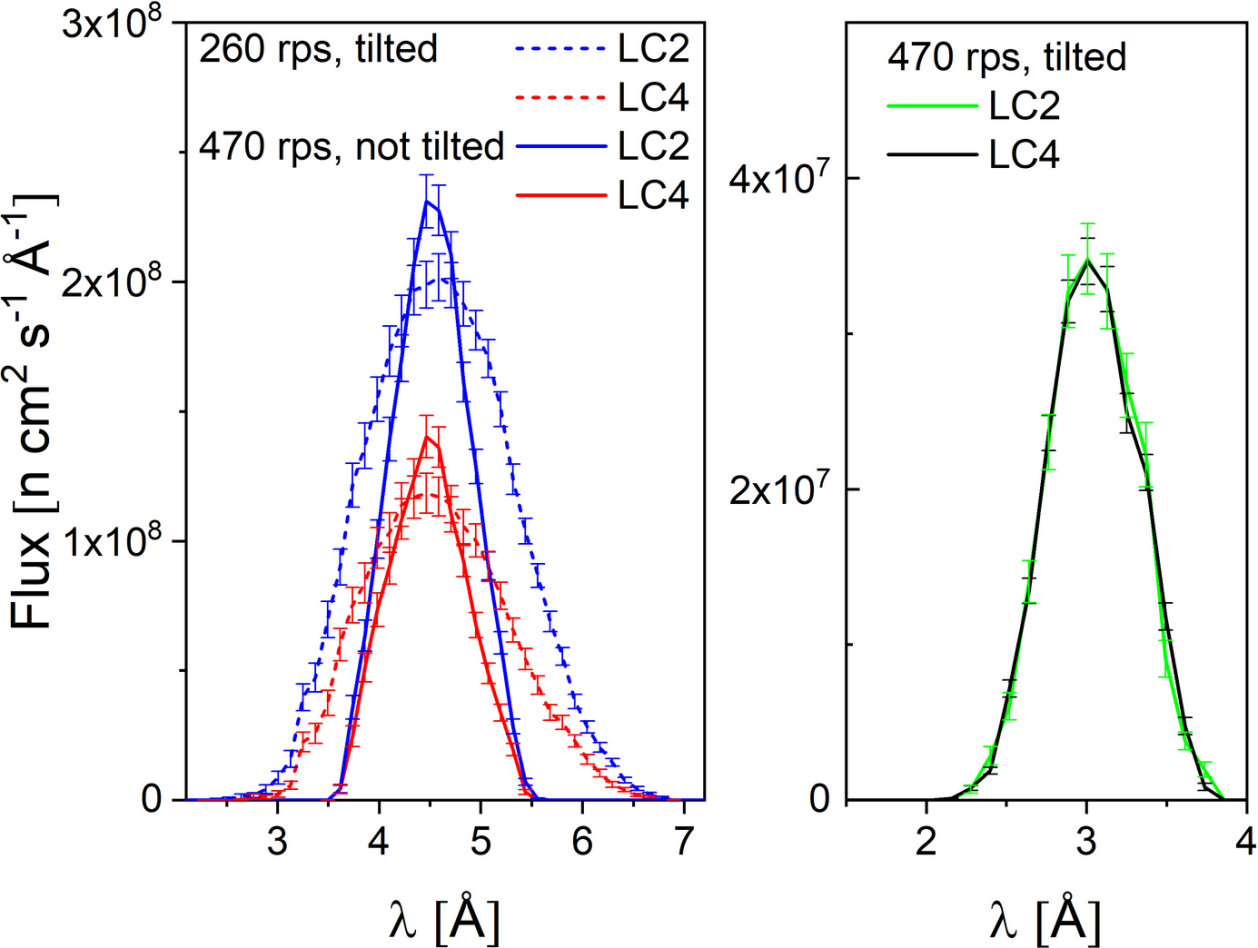
Simulated wavelength distribution of the neutron flux at the sample position of KWS-2 for different collimation lengths *L*_C_ with a ‘20%’ selector running with different speeds in different positionings with respect to the beam axis (see the text for detailed explanation).

Interestingly, when a low-resolution selector (having 36 instead of 72 twisted blades) is used, the shift in the central wavelength of the triangular distribution observed for very short wavelengths, just above the guide cut-off, provides a monochromatic beam with λ = 3 Å and Δλ/λ = 20.5% instead of λ = 2.8 Å and Δλ/λ = 14% in the case of the higher-resolution selector (Fig. 5 ▸). This is again an effect of the guide cut-off on the distribution of monochromated neutrons, depending on the monochromatization conditions. Regarding the neutron flux available at the sample position, for very short wavelength neutrons of λ = 2.8–3 Å, there is no difference between the *L*_C_ = 2 m and *L*_C_ = 4 m conditions (Fig. 6 ▸), as happens for the longer-wavelength neutrons, λ > 4.5 Å. Theoretically, on changing the collimation length between *L*_C1_ and *L*_C2_ the flux at the sample position varies with (*L*_C1_/*L*_C2_)^2^, while maintaining the same entrance and sample aperture size. However, for the case of shorter collimation lengths, the effect of the beam divergence plays an important role (Schwahn *et al.*, 1991 ▸). Shorter-wavelength neutrons are characterized by narrower divergences, as shown by equation (1). Thus, after they exit the S-guide the first collisions with the horizontal collimation guide happen in the vicinity of the collimation entrance. This smooths out the asymmetric vertical beam distribution and filters neutrons arriving at higher angles than the critical angle of the collimation guide segments (Radulescu *et al.*, 2012*b* ▸). Higher beam divergences cannot reach the sample due to the narrow sample aperture.

Therefore, only lower beam divergences are transported further up to the sample position, which means that the very short wavelength neutrons reaching the sample should experience a very small number of further collisions with the collimation guide, after entering the collimation system. For neutrons of λ = 2.8–3 Å that arrive at the sample position, this apparently happens at longer collimation lengths *L*_C_ > 4 m, which explains the unchanged neutron flux values as observed in Fig. 6 ▸.

Another observation from Figs. 5 ▸ and 6 ▸ is the difference between the maximum intensity with the selector tilted and not tilted for the same λ, which relates to the difference in selector transmission in the two cases. According to the producer’s specifications, the transmission at zero beam divergence decreases by about 10% when the selector is tilted at the angle ξ_i_ = −10° to the beam axis. Thus, using a velocity selector providing a relaxed Δλ/λ resolution, one can minimize the loss in flux at the sample position due to the missing CNS.

**Figure 7:**
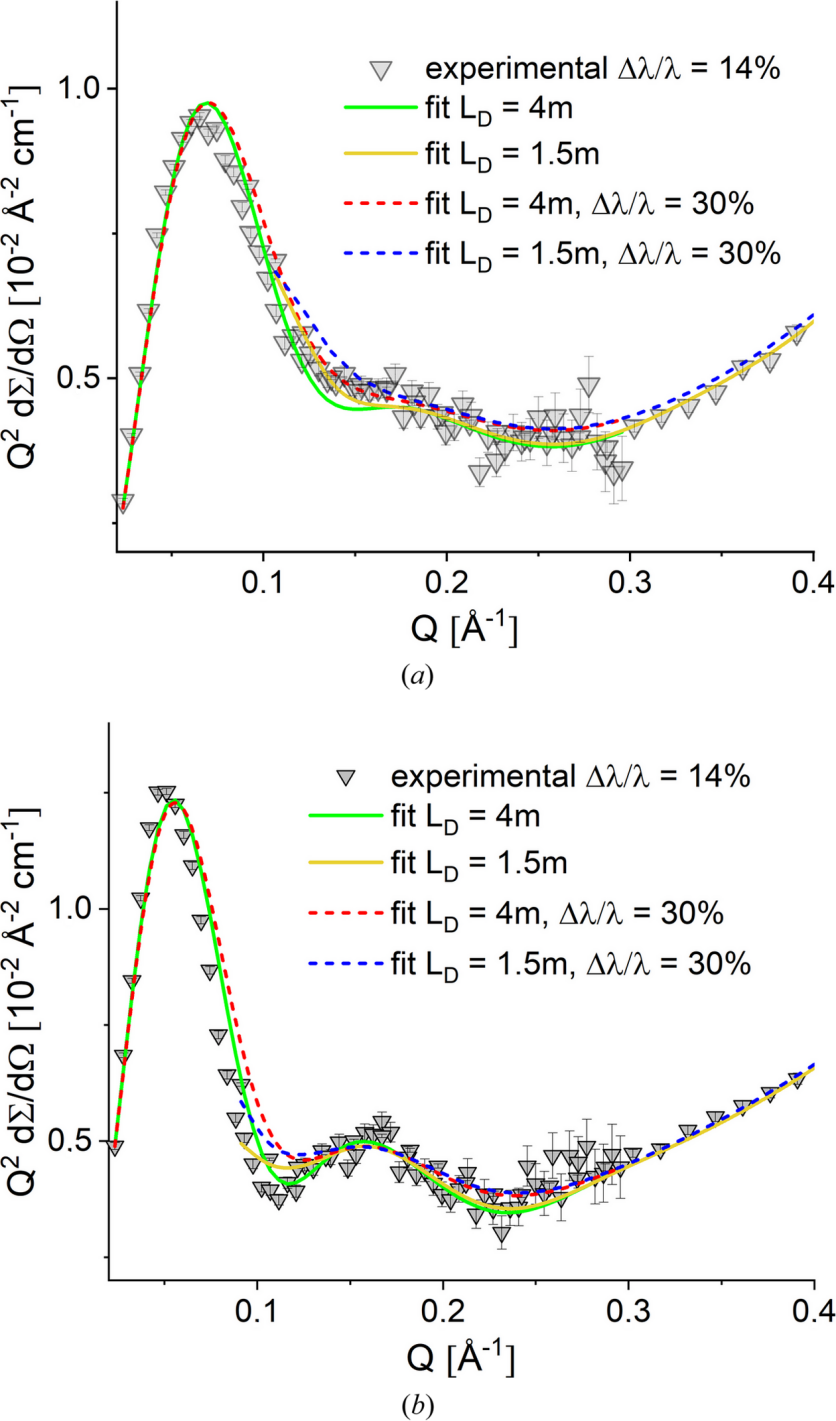
Kratky plots of the experimental data and fitted SANS curves of BSA (*a*) and ADH (*b*) proteins in buffer solution. The fitted curves indicated separately for data measured at different detection distance *L*_D_ were convoluted with different experimental resolution as indicated in the figure legend. BSA and ADH form factors were calculated according to the respective PDB structures 4f5s (Bujacz, 2012 ▸) and 4w6z (Raj *et al.*, 2014 ▸).

The effect of the relaxed Δλ/λ on the quality of the measured data was checked on the BSA and ADH protein solutions. Figs. 7 ▸(*a*) and 7 ▸(*b*) show in a Kratky-type presentation the scattering patterns of the two proteins at 5 mg ml^−1^ in buffer solution after corrections for the instrument, quartz cuvette and buffer solvent contributions were applied.

The scattering features of the two proteins in solution are clearly observed in the scattering patterns: the first bell-shaped maximum observed between 0.05 and 0.1 Å^−1^ denotes the size of the well folded globular shape proteins, while the shoulders observed at 0.15–0.2 Å^−1^, much more visible in the case of ADH due to its tetrameric structure, are an indication of the protein shape and multi-domain character of the proteins. The data measured at two different *L*_D_ were fitted simultaneously with the form factor calculated according to the respective Protein Data Bank (PDB; https://www.rcsb.org/) structures by using the computational code *Jscatter* (Biehl, 2019 ▸) with the data smeared according to Pedersen *et al.* (1990 ▸), taking into account the wavelength spread Δλ/λ = 14% (Radulescu, 2024 ▸) and the geometry of the instrument for the two selected detector distances. A good agreement between the calculated structure, the green and yellow solid lines in Figs. 7 ▸(*a*) and 7 ▸(*b*) for *L*_D_ = 4 and 1.5 m, respectively, and measured data was found with regard to the main scattering features.

Convoluting the calculated form factors with an instrument resolution based on larger Δλ/λ would still provide a good fit of the experimental data with a description of the main scattering features, as indicated by the red and blue dashed lines in Figs. 7 ▸(*a*) and 7 ▸(*b*). These were obtained by considering Δλ/λ = 30% being delivered by a tilted low-resolution velocity selector. Thus, a gain in intensity while using a low-resolution velocity selector when working with the KWS-2 SANS diffractometer in the TNS regime of the FRM II reactor would be feasible, with good data quality for many of the typical topics studied at the instrument. Of course, for experiments demanding a higher resolution for observing specific scattering features, a compromise between the intensity on the sample and resolution should be found.

**Figure 8:**
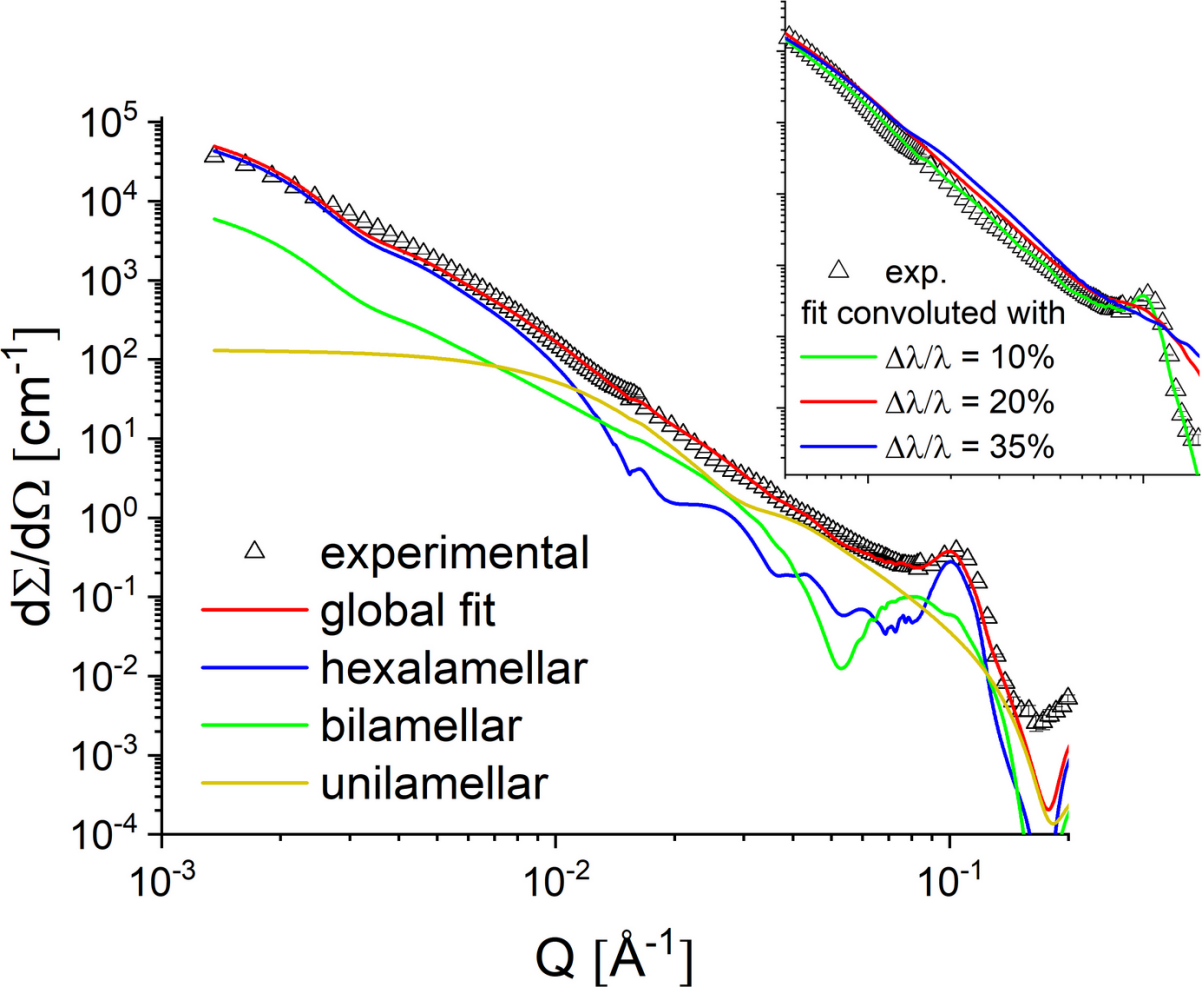
SANS of DPPC–MPOx2 (symbols) at 30 mg ml^−1^ in D_2_O at 25°C. The combined contributions of unilamellar (yellow), bilamellar (green) and hexalamellar (blue) vesicles to the SANS fitted curve (red) are shown. The inset displays the effect of different Δλ/λ resolution values on the global fit results: the sharp correlation peak attributable to the hexa­lamellar vesicles is barely observable when Δλ/λ ≥ 20%.

The performance limit of the proposed low-resolution SANS for enhancing the intensity on the sample was verified on the DPPC–MPOx2 system, which is characterized by vesicles with variable lamellarity in D_2_O. Due to the variable hydro­phobicity, the MPOx polymers interact in different ways with the hydro­phobic interior of the DPPC bilayers, leading to multilamellar formulations in solution whose scattering is characterized by the appearance of sharp correlation peaks at high *Q*. The research on DPPC–MPOx systems has been reported in detail elsewhere by Papagiannopoulos *et al.* (2021 ▸). Here we examine the impact of lower instrumental resolution on the quality of the data. Fig. 8 ▸ shows the experimental results measured at KWS-2 with λ = 4.5 Å and Δλ/λ = 10%, together with the model fitting results convolved with the instrumental resolution, and the effect of relaxing the resolution of Δλ/λ on the global fitting results (inset in Fig. 8 ▸).

The model of DPPC vesicles (uni- and bilamellar vesicles) was superimposed with a model for multilamellar vesicles to capture the scatter at low *Q* and the sharp peak at high *Q*. The best fit was achieved by superimposing a hexalamellar vesicle model. A detailed report on the structure and density parameters obtained from the fitting procedure and their interpretation has already been published by Papagiannopoulos *et al.* (2021 ▸).

Interestingly, the sharp peak observed at high *Q* for Δλ/λ = 10% would disappear if an instrumental resolution of Δλ/λ = 20% or 35% were considered, with a smoother scattering profile being measured at high *Q* in these cases. Therefore, maintaining a high resolution of Δλ/λ = 10% would be essential for the thorough characterization of systems with highly lamellar characteristics, meaning that such investigations in the TNS regime would only be possible with KWS-2 if a significantly longer acquisition time than required with the standard CNS configuration were considered.

#### 4.2.2. Intensity enhancement by focusing lenses

To improve the resolution (*Q*_min_) and intensity on the sample, several types of focusing elements have been proposed or developed over time, including compound refractive lenses (Choi *et al.*, 2000 ▸; Koizumi *et al.*, 2007 ▸; Frielinghaus *et al.*, 2009 ▸; Iwase *et al.*, 2011 ▸), mirrors (Alefeld *et al.*, 2000 ▸), magnetic lenses (Kumada *et al.*, 2023 ▸) and Wolter optics (Liu *et al.*, 2012 ▸; Wu *et al.*, 2021 ▸; Samothrakitis *et al.*, 2024 ▸).

The KWS-3 at JCNS (Goerigk & Varga, 2011 ▸) is the only mirror focusing instrument in operation today. Although many attempts have been made in the past to use this technique for SANS experiments, these failed due to the lack of availability of high-quality mirror reflecting surfaces. KWS-3 offers the advantage of avoiding gravitational effects associated with focusing with highly chromatic composite lenses when a wide Δλ/λ is used, but it also has disadvantages in some cases in terms of sample transmission and intensity due to the long wavelength used, λ = 12 Å. Instruments based on asymmetric Wolter mirrors have been proposed (Liu *et al.*, 2012 ▸; Wu *et al.*, 2021 ▸; Samothrakitis *et al.*, 2024 ▸), but again they require the use of longer wavelengths λ than pinhole SANS.

Recently, it has also been shown how Fresnel zone plates of nickel and silicon can be used to improve resolution (*Q*_min_) in SANS experiments, illustrating the promising potential of neutron diffraction optics (Dhanalakshmi Veeraraj *et al.*, 2025 ▸). However, this early concept is still hampered by a high background, a problem that needs to be solved before actual application can be tested. Other older attempts to build Fresnel lenses (Adachi *et al.*, 2004 ▸) were also unsuccessful, as the processing of the lenses led to unwanted refraction effects. Magnetic focusing (Kumada *et al.*, 2023 ▸), in which there is no material in the beam so that there is no background due to diffuse scattering from the lens material, offers a certain advantage. The disadvantage is that this focusing method requires polarized neutron beams where one spin state is focused while the other spin state is defocused, which reduces the efficiency to a maximum of 50%.

Compound refractive lenses are commonly used in SANS beamlines. The focusing of cold neutrons with several lenses was first proposed by Choi *et al.* (2000 ▸): with biconcave MgF_2_ lenses, the *Q* range on a SANS pinhole instrument was extended down to ∼0.004 Å^−1^. As described in detail by Eskildsen *et al.* (1998 ▸), the refractive index for cold neutrons is less than one, which is why neutron lenses must be concave. Several elements and isotopes suitable for neutron optics are listed by Eskildsen *et al.* (1998 ▸), where materials such as MgF_2_, BeO or crystalline CO_2_ are discussed as potentially suitable for neutron lenses. In contrast to some other materials, MgF_2_ is readily available, cheap and easy to process. The focal length of such a lens is usually very long, about 100–200 m. Therefore, for a SANS instrument where typically *L*_D_ extends to 15–20 m, several such lenses should be used in series, depending on the neutron wavelength λ used. Generally, the package of MgF_2_ neutron lenses focuses the image of the entrance aperture, placed on one focal point of the lens system, on the detector which is placed on the other focal point of the lenses.

One can distinguish two operation modes of focusing lenses, depending on the scientific goal.

First, working with a small entrance (collimation) aperture at *L*_D_ = 20 m, a small direct beam spot can be focused on a high-resolution detector (HRD) downstream of the sample, thus allowing neutrons scattered at much lower scattering angles than in pinhole mode to be detected. This provides optimal conditions for reaching a lower value for the minimum wavevector transfer *Q*_min_ than in conventional pinhole mode. Usually *Q*_min_ ≈ 1 × 10^−4^ Å^−1^ can be reached in this experimental configuration, a factor 10 better in *Q*_min_ than in the pinhole mode. At KWS-2, 26 MgF_2_ parabolic lenses of diameter ϕ = 50 mm are used in the configuration λ = 7 Å/*L*_C_ = 20 m/*L*_D_ = 17 m together with a scintillation HRD (Radulescu *et al.*, 2012*a* ▸). Other SANS instruments use spherical lenses and HRD (scintillation) to reach *Q*_min_ ≈ 0.0001 Å^−1^: SANS-J of JAEA (Kumada *et al.*, 2023 ▸) with 70 lenses of diameter ϕ = 15 mm, and SANS-U of Tokyo University (Iwase *et al.*, 2011 ▸), with 55 lenses of diameter ϕ = 30 mm, both operating at the JRR-3 reactor in Tokai, Japan. Such low *Q*_min_ values are reached at other instruments using either a multi-hole collimation system together with a combination of MgF_2_ lenses and gravity-canceling prisms combined with a scintillation HRD, like at the VSANS instrument at NIST (Barker *et al.*, 2022 ▸), or a converging multiple vertical slit collimation system which focuses the direct beam on a scintillation HRD, like at the MS-VSANS instrument at CSNS, Dongguan, China (Zuo *et al.*, 2024 ▸). Both instruments provide a *Q*_min_ down to 1 × 10^−4^ Å^−1^. The additional complexity of these instruments related to either the converging beam collimation or the data smearing while measuring in slit geometry can be studied in detail in the respective publications (Barker *et al.*, 2022 ▸; Zuo *et al.*, 2024 ▸).

Secondly, focusing lenses can be used for enhancing the intensity on the sample. When a larger size of the entrance aperture is used, as for the conventional pinhole mode, the beam can be focused on the main SANS detector and captured on the large beamstop that is typically used in this mode, thus maintaining the *Q*_min_ resolution as for the pinhole configuration. The advantage of this second working mode with focusing lenses is that the beam size on the sample can be increased up to a size that corresponds to the lens size. This will thus allow the use of a much higher intensity for the measurement, with a significant advantage over the pinhole mode while using the same entrance aperture size and beamstop size. The challenge while working in this mode is the availability of a larger sample amount than in the standard pinhole mode, to fill a larger size cuvette that can be exposed to the large beam size. However, this working mode is meant for the low-*Q* measurements on soft-matter samples such as gel, colloidal or micellar systems or membranes, films and powders, which require a thorough structural characterization at a larger length scale. Such samples can be provided in a sufficient amount to fulfill the requirements of this demanding sample geometry. Moreover, contrast-variation SANS on samples in liquid state would benefit from this mode, when enhancement of intensity on the sample would provide better measurement statistics for such weak scattering conditions over a typical acquisition time.

The KWS-2 SANS diffractometer is equipped with 26 MgF_2_ parabolic lenses with a diameter of ϕ = 50 mm. To focus monochromatic beams of different wavelength λ, as defined by the entrance (collimation) aperture, onto the detector positioned at *L*_D_, a different number of lenses is required for different λ values. Thus, 4 lenses are used for λ = 17.5 Å, 10 lenses for λ = 10 Å and 26 lenses for λ = 7 Å. With an entrance aperture of 50 × 50 mm at a collimation length *L*_C_ = 20 m, the lenses can focus a direct beam spot of the same size as in the pinhole SANS method onto the main SANS detector, so that the same resolution (*Q*_min_) as in pinhole SANS can be achieved. For ideal focusing, the condition *L*_1_ = *L*_2_ should be fulfilled, although *L*_2_ < *L*_1_ may also work with appropriate adjustment of the source image, namely, the size of the entrance aperture (Dahdal *et al.*, 2014 ▸). The neutron transmission of lenses is influenced by processes such as neutron absorption and scattering on the lens material. To minimize the lens scattering that is mainly caused by thermal vibrations in the lens material (phonons), the lenses must be cooled to 77 K (Frielinghaus *et al.*, 2009 ▸). Geometric aberration correction by the parabolic shape together with the chromatic aberrations due to the wide Δλ/λ used are discussed by Frielinghaus *et al.* (2009 ▸).

To quantify the intensity enhancement on the sample using lenses and large beam size, polystyrene size standards (*R*_ps_ = 150 Å) in D_2_O solution were measured with different beam sizes on a large sample and with all 26 cold lenses in beam. The size of the entrance aperture at *L*_C_ = 20 m was 50 × 50 mm, as in the standard pinhole mode, and the main SANS detector with a beamstop of size 70 × 70 mm was positioned at *L*_D_ = 17.5 m.

**Figure 9:**
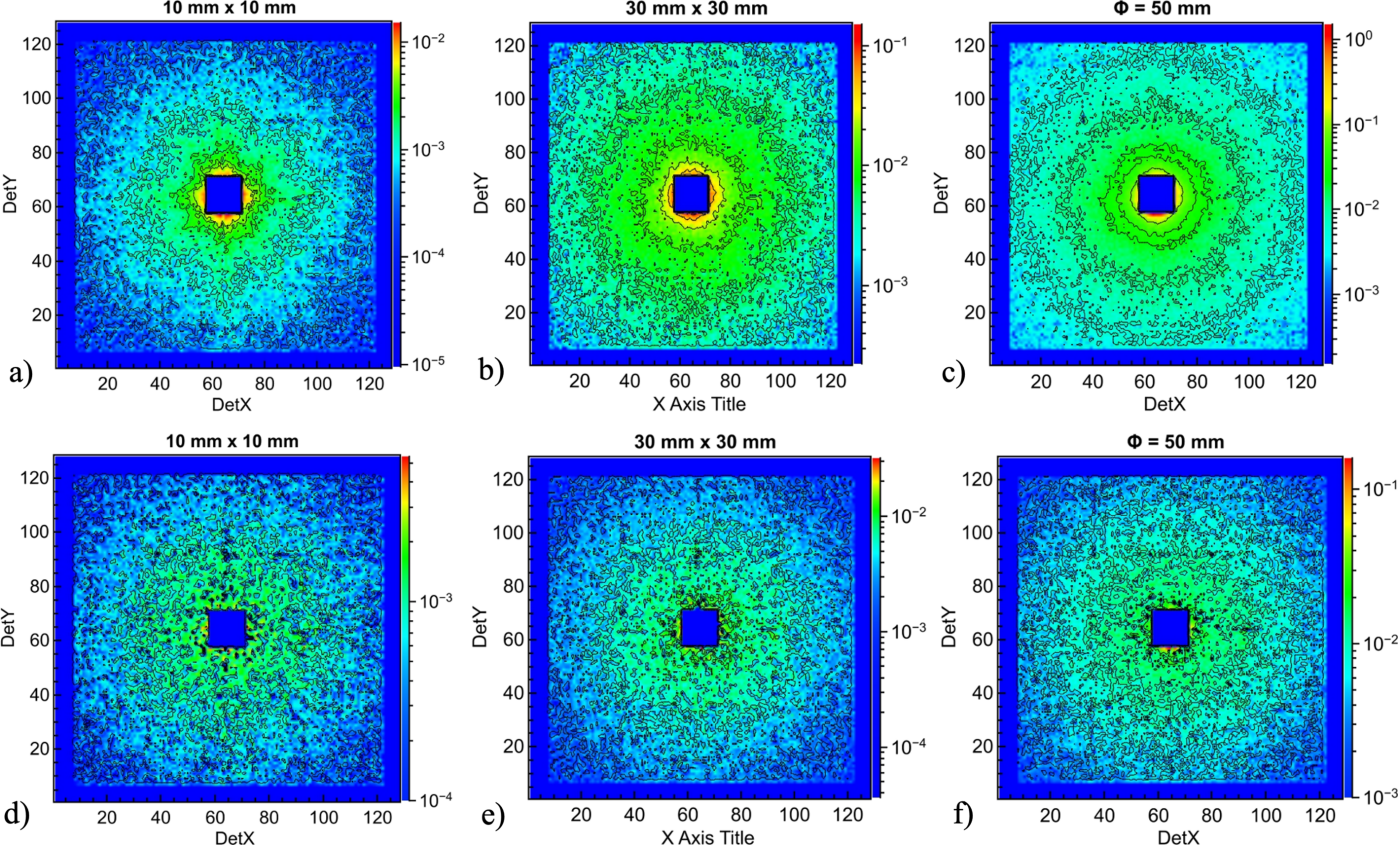
2D scattering patterns from polystyrene particles in solution measured in focusing mode at KWS-2 with 26 MgF_2_ lenses using different sample aperture size, as indicated at the top of each panel, *i.e.* different sample size. Panels (*a*–*c*) show the results from particle solution in the quartz cuvettes while panels (*d*–*f*) present the results after the correction for the quartz cuvette contribution was applied.

**Figure 10:**
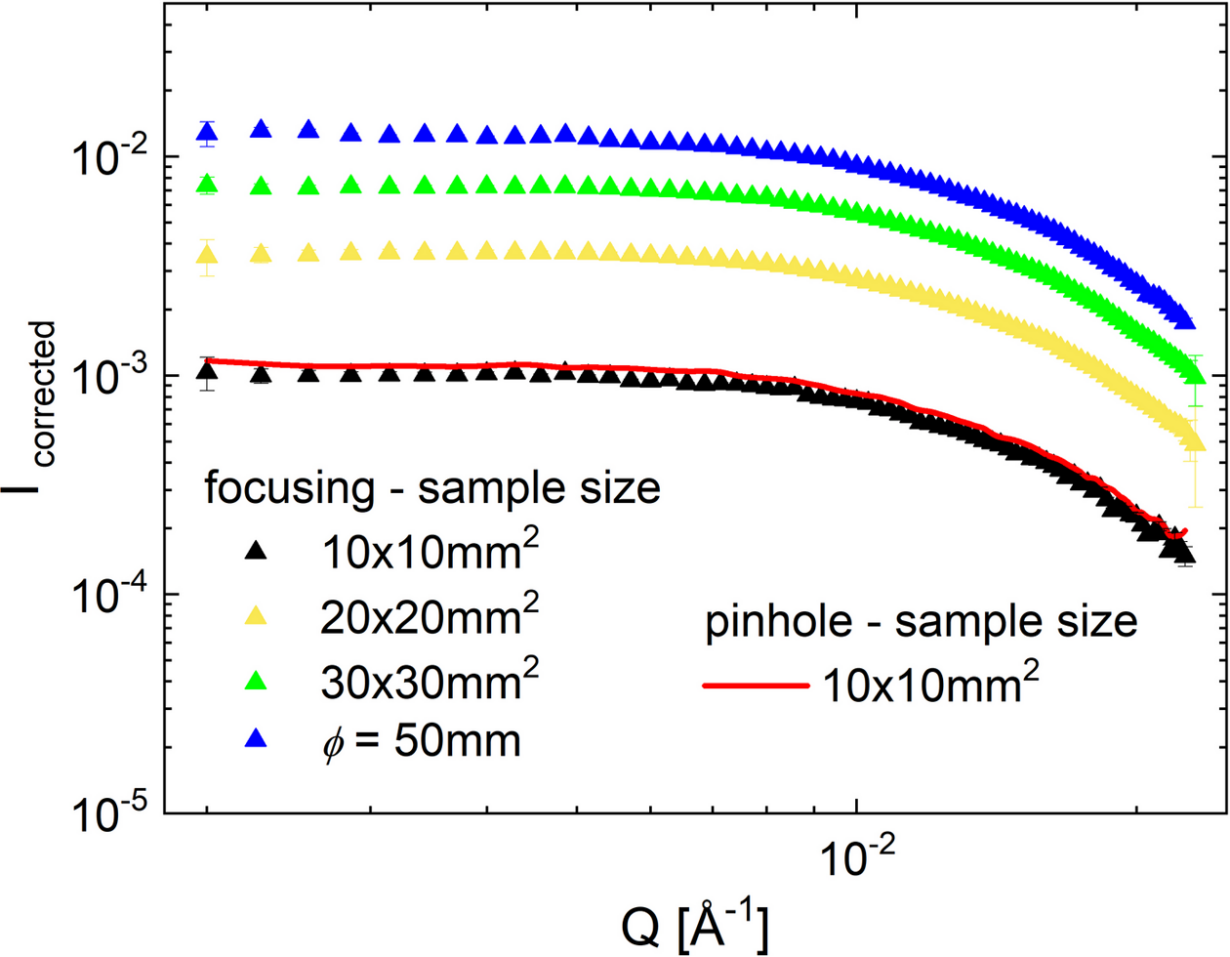
The corrected and normalized 1D scattered intensity from polystyrene particles in solution as measured in different working modes at KWS-2. The red solid line shows the result measured in pinhole geometry while the full symbols report the results measured in focusing mode with 26 MgF_2_ lenses using different sample aperture size, *i.e.* different sample size.

Fig. 9 ▸ presents the 2D SANS patterns collected for the sample in quartz cuvettes using a square beam of size 10 × 10 mm (panel *a*) and 30 × 30 mm (panel *b*) and a circular beam of size (diameter) ϕ = 50 mm (panel *c*) on the sample. The data corrected for the empty cuvette contribution are shown in panels (*d*), (*e*) and (*f*), respectively. The corrected scattering patterns look qualitatively very similar to those measured in pinhole geometry, without any spurious features. The corrected and normalized 1D scattered intensity for different beam sizes considered is shown in Fig. 10 ▸.

For a quantitative estimation of the lens enhancement of the intensity on the sample, we rely on the well known formula used in correction and calibration,
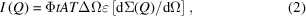
where Φ represents the flux on the sample, *t* the sample thickness, *A* the sample area exposed to the beam, *T* the sample transmission, ɛ the detector efficiency and ΔΩ the solid angle subtended by one pixel of the detector (Heenan *et al.*, 1997 ▸; Allen, 2019 ▸).

The main goal of a SANS experiment is to determine 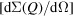, which is a property of the sample of interest and delivers the main structural information about the sample when interpreted with appropriate models. If we consider now equation (2) for two different experimental conditions, measurement in pinhole geometry and in focusing mode with lenses, and we keep all the geometric parameters constant with a sample aperture of 10 × 10 mm, and hence the beam size on the sample too, the only parameter that influences the measured *I*(*Q*) is the flux Φ. For the lens configuration this can be expressed as Φ_lens_ = *T*_lenses_Φ_pinhole_, with *T*_lenses_ the transmission of the lens package for that particular beam size. Thus, dividing the measured intensities in these two experimental conditions, according to equation (2), the transmission of the lens package for a 10 × 10 mm lens or beam size used in the experiment is obtained. Furthermore, assuming that Φ is homogeneously distributed at the sample position, by increasing the lens size the intensity on the sample will be proportional to *A* – the sample area exposed to the beam – and the *T*_lenses_ that must be considered for each lens size used. Using the same approach, *T*_lenses_ for each lens size configuration can be obtained.

**Figure 11:**
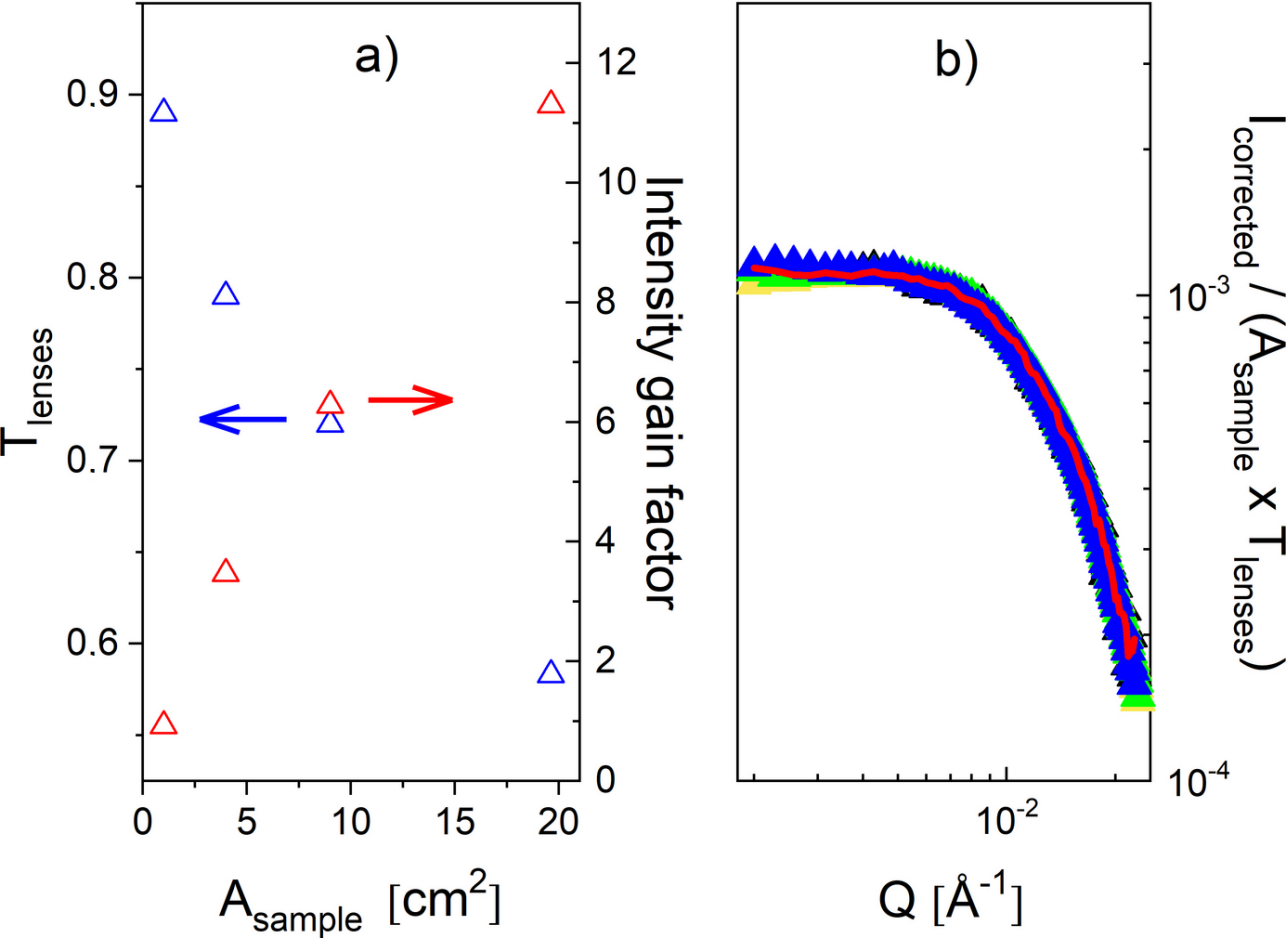
Measured transmission using the 26 cold lens package at KWS-2 (blue symbols) and intensity gain factor by increasing the beam size on a large sample in focusing mode as a function of the sample area in beam – left panel. The corrected and normalized 1D scattered intensity from polystyrene particles in solution as measured in focusing mode with 26 MgF_2_ cold lenses using different sample aperture size, *i.e.* different sample size, after normalization to the sample area and transmission of the lens package – right panel.

The lens transmission *T*_lenses_ for each beam size considered, *i.e.* sample size, is shown in Fig. 11 ▸(*a*) as blue symbols, while the factor for the increase in intensity when increasing the lens size, *i.e.* the sample size, is shown as red symbols. Normalizing the scattering patterns to the product of sample area and lens transmission results in all scattering curves lying on top of each other [Fig. 11 ▸(*b*)], which proves the validity of the approach used in this experiment. According to equation (8) of Frielinghaus *et al.* (2009 ▸), for a package of 26 lenses held at 77 K, a transmission of approximately *T*_lenses_ = 0.6 is theoretically calculated when the full lens size is used in the experiment, which is very close to the measured *T*_lenses_ = 0.585. The measured sample transmission *T*_sample_ was always 0.86, independent of the lens size, *i.e.* the beam size on the sample.

Thus, an intensity increase by a factor of about 11 is achieved when using the focusing mode on the KWS-2 by increasing the sample size to a diameter of up to 5 cm while maintaining the same *Q*_min_ as in pinhole mode. This gain in intensity on the sample can thus compensate for the decrease in intensity for λ ≥ 7 Å when working only with the TNS at the FRM II reactor compared with standard reactor operation with a CNS.

For practical reasons, only focusing experiments with λ = 7 and 10 Å have been carried out at KWS-2 so far, so that either 26 lenses or 10 lenses were used in the beam focusing. The focusing of neutrons with λ = 17.5 Å with 4 lenses served mainly for methodological development (Radulescu *et al.*, 2023 ▸). Moreover, neutrons with λ > 7 Å cannot be delivered with the inclined Airbus selector due to resonance effects that prohibit the use of the velocity selector at lower rotational speeds. Therefore, these cases are not of practical significance for KWS-2.

## 5. Conclusions

The performance of KWS-2 was evaluated by measurements and *McStas* simulations under the condition that only the TNS is available at the FRM II reactor, and the results were compared with the established performance with the CNS.

**Figure 12:**
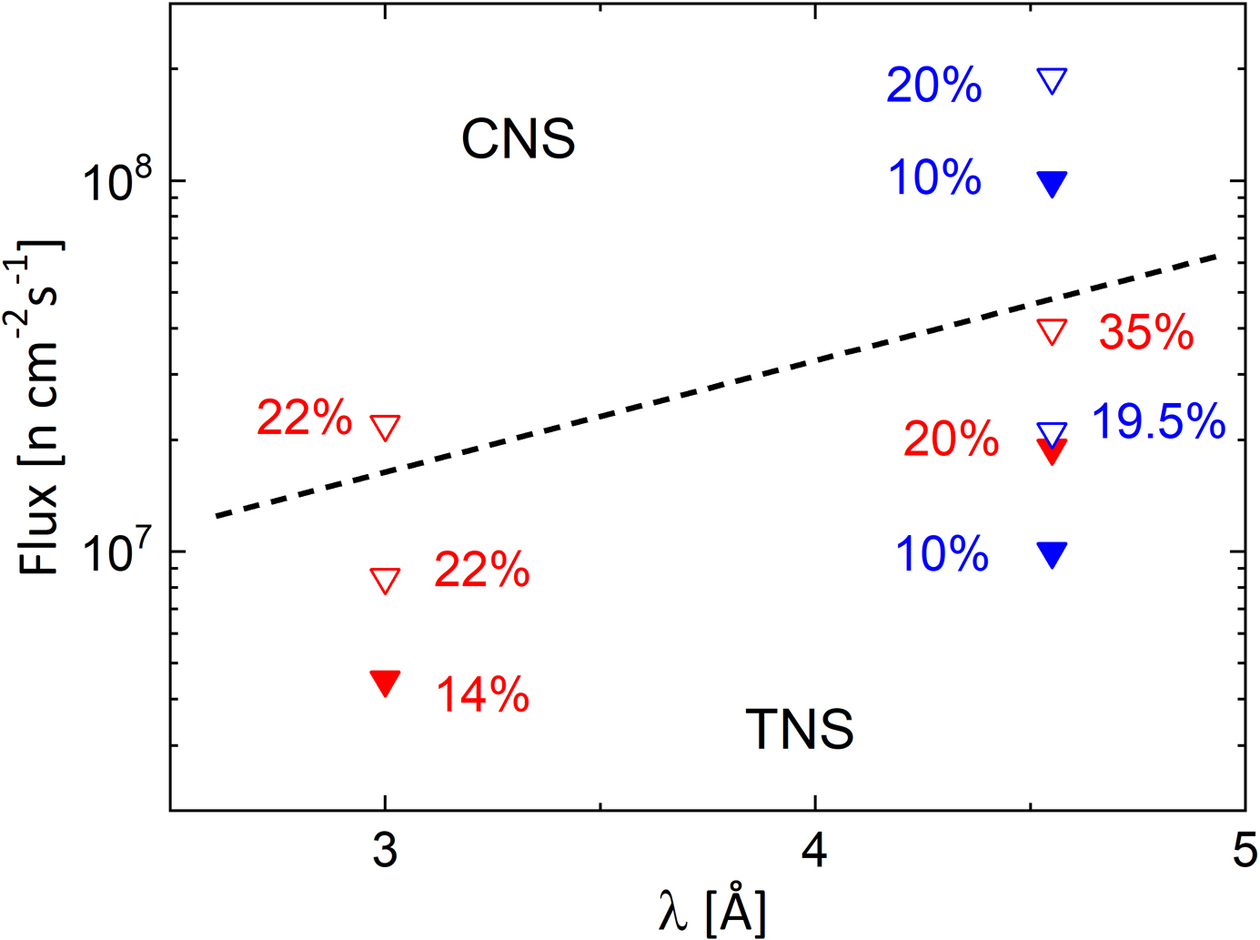
Summary of the results of the *McStas* simulations of the maximum flux at the sample position of KWS-2 for the various operating conditions considered. The broken line delimits the values obtained with the CNS or the TNS in operation. The open symbols represent the results with a ‘20%’ velocity selector, while the filled symbols are for a ‘10%’ velocity selector. The blue symbols show the results obtained with the selectors operating parallel to the beam axis, while the red symbols represent the selectors operating in an inclined position.

The expected flux decrease for different wavelengths λ when operating the reactor with TNS only compared with the standard operation with CNS as well as the improvements of the flux values when using different types of velocity selectors are summarized in Fig. 12 ▸. When the FRM II reactor is operated with TNS only, the decrease in flux is about 2.5-fold for short-wavelength neutrons around 3 Å and about 10-fold for neutrons with λ ∼ 4.5 Å compared with CNS operation mode, while maintaining the same velocity selector settings as in standard operation mode (Houston *et al.*, 2018 ▸). The loss factor is even higher at longer wavelengths (Table 1 ▸). According to the measurements, relaxing the resolution Δλ/λ to reduce the flux decrease to only about a factor of 4 for λ ∼ 4.5 Å would still be beneficial for the structural characterization of small biological morphologies such as proteins and protein–polymer complexes. This would be one of the most studied topics if only thermal flux neutrons were available at FRM II. However, lamellar effects, which lead to sharp correlation peaks in the high-*Q*-value range, are barely observable in measurements with a relaxed resolution of Δλ/λ ≥ 20%.

Furthermore, by using MgF_2_ focusing lenses when working with large samples, an increase in intensity on the sample of up to 11-fold is achieved while maintaining *Q*_min_ resolution as with the standard pinhole mode, restoring most of the flux loss expected for λ ≥ 7 Å when only the TNS is in operation at the FRM II reactor. A summary of the simulated flux values at the sample position while working either with cold or with thermal neutrons, as well as the reduction factors depending on the experimental configuration, source and velocity selector, is presented in Table 1 ▸.

## Figures and Tables

**Table 1:** Summary of simulated intensity values (n s^−1^) at the sample position of KWS-2 for CNS and TNS modes for different configurations (neutron source, selector type, beam size) Columns 2 and 3 show the intensities in the standard pinhole geometry with a beam size of 1 cm^2^, while column 4 indicates the reduction factors of the intensity when working with thermal neutrons compared with cold neutrons. The intensity obtained with thermal neutrons by further loosening the resolution (column 5) or increasing the beam size in focusing mode (column 7) is also indicated, with the corresponding expected reduction factors (columns 6 and 8).

λ (Å)	CNS, 20% (1 cm^2^)	TNS, 20% (1 cm^2^)	Reduction factor TNS versus CNS	TNS, 35% (1 cm^2^)	Reduction factor TNS 20% versus 35%	TNS, 20%, focusing (19.625 cm^2^)	Reduction factor TNS (with lenses) versus CNS
3	2.2 × 10^7^	8.5 × 10^6^	2.6	–	–	–	–
4.5	2.0 × 10^8^	2.0 × 10^7^	10	4.8 × 10^7^	4.2	–	–
7	1.2 × 10^8^	6.5 × 10^6^	18.5	1.2 × 10^7^	10	7.2 × 10^7^	1.66
10	4.0 × 10^7^	1.2 × 10^6^	33.3	–	–	1.3 × 10^7^	3
